# Pleiotropic Effects of Calcium Channel Blockers

**DOI:** 10.1007/s11906-012-0269-4

**Published:** 2012-05-18

**Authors:** R. Preston Mason

**Affiliations:** Cardiovascular Division, Department of Medicine, Brigham and Women’s Hospital, Harvard Medical School, PO Box 7100, 100 Cummings Center, Suite 135L, Beverly, MA 01915 USA

**Keywords:** Calcium channel blockers, Endothelial function, Nitric oxide, Central aortic pressure, Statins, Pleiotropic effects, Mechanisms, Antihypertensive agents

## Abstract

Clinical trials have reported reduced cardiovascular events with certain antihypertensive agents at a rate that could not be predicted by changes in brachial arterial pressure alone. These findings may be explained, in part, by pleiotropic effects of these agents and modulation of central blood pressures. This review focuses on the mechanisms by which calcium channel blockers exert pleiotropic effects, both alone and in combination with statins and inhibitors of the renin-angiotensin system. The essential role of nitric oxide (NO) in maintaining endothelial function and the relationship between NO and reactive oxygen species are discussed in the context of the etiology of hypertension. The importance of managing global cardiovascular risk is emphasized, as hypertension commonly clusters with dyslipidemia and loss of glucose control. From a mechanistic viewpoint, these risk factors contribute to endothelial dysfunction, oxidative stress, and inflammation in a synergistic fashion. A greater understanding of the mechanisms of actions of these cardiovascular agents may lead to more effective drug combinations, to the benefit of individual patients. Furthermore, by elucidating the biological mechanisms by which cardiovascular risk factors lead to vascular injury, we may highlight common pathways and identify novel therapeutic targets.

## Introduction

Blood pressure is a primary and independent risk factor for cardiovascular disease [1]. By convention, blood pressure is measured using a sphygmomanometer (manual or automatic) over the brachial artery. The vast majority of the evidence supporting the benefits of blood pressure reduction is based on brachial blood pressure measurements [2].

Over the last 10 years, several antihypertensive drug treatment trials have shown an unexpected discrepancy between reduction in brachial blood pressure and observed clinical outcomes. In the Heart Outcomes Prevention Evaluation (HOPE) [3], the Losartan Intervention For Endpoint reduction (LIFE) in hypertension [4], and the Australian National Blood Pressure 2 (ANBP2) [5] trials, the observed clinical benefits tended to be greater than those expected from the decrease in brachial blood pressure. This may be explained by the pleiotropic effects of the antihypertensive drugs used in these trials. In particular, the beneficial effects of these agents on endothelial function and its vascular manifestations, such as changes in central aortic pressure and atheroma development.

It has been proposed that central aortic pressure may have more pathophysiological relevance than peripheral blood pressure as a marker for the development of cardiovascular disease [6•, 7]. Central aortic pressure is determined by the combination of cardiac output and peripheral vascular resistance that is modulated by arterial stiffness along with the timing and magnitude of pressure wave reflections in the arterial tree. The pressure wave generated by the left ventricle during cardiac systole travels through the vessels until it reaches the small muscular arteries and arterioles where it is reflected [8]. The pressure waveform at any point in the arterial tree is therefore the sum of both forward and backward waveforms. When the large arteries are healthy and compliant, the reflected wave merges with the forward wave in the proximal aorta during diastole, augmenting diastolic blood pressure and aiding coronary perfusion. However, when the large arteries are stiff, pulse wave velocity increases, accelerating both incident and reflected waves [8]. This results in the reflected wave merging with the incident wave in systole, thus augmenting central aortic systolic rather than diastolic pressure. Thus, central aortic stiffness contributes directly to a wide pulse pressure with higher systolic and lower diastolic blood pressure. Furthermore, the state of the vasculature in the peripheral circulation also affects the proportion of the incident wave that is reflected, thus affecting central pressure.

Observational studies have shown that the difference between brachial and central arterial pressures can vary by between 2 and 33 mm Hg [9, 10]. Furthermore, different antihypertensive drugs have been shown to have similar effects on brachial blood pressure, but different effects on central aortic pressure [6•, 11–13]. This may, in part, explain why central aortic pressure has been shown to have superior prognostic value with respect to cardiovascular events than brachial blood pressure in clinical studies [6•, 11–13].

The Conduit Artery Function Evaluation (CAFE) study [6•], was a substudy of the Anglo-Scandinavian Cardiac Outcomes Trial (ASCOT). In ASCOT, patients with hypertension and at least three additional cardiovascular risk factors were randomized to an atenolol plus bendroflumethiazide-based treatment regimen or an amlodipine plus perindopril-based regimen. The CAFE study recruited 2,199 ASCOT patients after the first year of ASCOT follow-up (mean age of the cohort at baseline, 63 years) and followed them for up to 4 years. Radial artery applanation tonometry and pulse wave analysis were used to derive central aortic pressures and hemodynamic indices. Throughout follow up, derived central aortic systolic pressure was substantially lower in the amlodipine-based treatment group compared with the atenolol-based treatment group (area under the curve [AUC] difference, 4.3 mm Hg; 95 % confidence interval [CI], 3.3 to 5.4; *P* < 0.0001), whereas brachial systolic blood pressure was similar between the two treatment arms (AUC difference, 0.7 mm Hg; 95 % CI, –0.4 to 1.7; *P* = 0.2). Furthermore, central pulse pressure (PP) was associated with total cardiovascular events and procedures and the development of renal impairment (unadjusted *P* < 0.0001; adjusted for baseline values *P* < 0.05). The CAFE study demonstrated that antihypertensive drugs can have different effects on central aortic pressure despite similar brachial blood pressure measurements. The authors of the CAFE study proposed that their study elucidated a plausible mechanism to explain the superior clinical outcomes observed in the amlodipine-based treatment arm of ASCOT. They also speculated that central aortic pressure measurements may provide an explanation for the differences observed in other major outcome trials including LIFE [4] and HOPE [3].

The findings of the CAFE study [6•] supported those of the Preterax in Regression of Arterial Stiffness in a Controlled Double-Blind Study (REASON) [13], which reported that peripheral blood pressure measurements did not accurately reflect changes in central aortic pressure following treatment with different antihypertensive drugs. The finding that central pressure and wave reflection indices are strong independent predictors of all-cause and cardiovascular mortality has also been demonstrated in high-risk patient groups, including those with end-stage renal failure (Table 1) [11, 12].

**Table 1:** Studies reporting differences in measured brachial and central blood pressure that may explain cardiovascular outcomes

Study	Measurements	Patient population	Key result
Strong Heart Study (2007, 2009) [7, 14]	Sphygmomanometer to measure brachial BP. Radial applanation tonometry to determine central BP	Population-based, longitudinal study among 3,520 American Indians followed for a mean of 4.8 years	Central PP was strongly associated with carotid intima-media thickness, plaque score, and vascular mass. Central PP was an independent predictor of CV outcomes
CAFE (2006) [6•]	Semiautomated oscillometric device to measure brachial BP. Radial artery applanation tonometry and pulse wave velocity analysis to derive central aortic pressure and hemodynamic indexes	Substudy of the ASCOT study. 2,199 patients with hypertension and ≥3 additional CV risk factors previously randomized to an amlodipine or atenolol-based regimen followed for up to 4 years	Central aortic systolic BP was lower in the amlodipine versus atenolol arm throughout follow-up (AUC difference 4.3 mm Hg, 3.3 to 5.4, *P* < 0.0001). Brachial systolic BP similar (AUC difference 0.7 mm Hg, –0.4, 1.7, *P* = 0.2). May explain lower CV events in amlodipine arm
REASON (2004) [13]	Sphygmomanometer to measure brachial BP. Pulse wave velocity analysis and pattern of wave reflections to derive central aortic pressure	375 patients with hypertension randomized to atenolol or perindopril + indapamide, followed for 1 year	Treatment with perindopril + indapamide decreased brachial and central systolic BP significantly more than atenolol. In the perindopril + indapamide group the difference between brachial and central systolic BP was 8.28 ± 1.53 mm Hg versus 0.29 ± 1.61 mm Hg in the atenolol group
Safar (2002) [12], London (2001) [11]	Aortic pulse wave velocity measurement and determination of arterial wave reflection by applanation tonometry on the common carotid artery	180 patients with end-stage renal failure followed for a mean of 4.3 years. 40 CV and 30 non-CV events occurred	Aortic pulse wave velocity, increased augmentation index and carotid PP, were independent predictors of all-cause and CV mortality

*ASCOT* Anglo-Scandinavian Cardiac Outcomes Trial; *AUC* area under the curve; *BP* blood pressure; *CAFE* Conduit Artery Function Evaluation; *CV* cardiovascular; *PP* pulse pressure; *REASON* Preterax in Regression of Arterial Stiffness in a Controlled Double-Blind Study

These findings are complemented by the results of the Strong Heart Study [7, 14], a population-based, longitudinal study among 3,520 American Indians (mean age 58 years) followed for a mean of 4.8 years. This study reported that a central PP greater than 50 mm Hg and not brachial PP was an independent predictor of cardiovascular outcomes, regardless of age, sex, or diabetes. Furthermore, central PP was strongly associated with carotid intima-media thickness, plaque score, and vascular mass, and was a stronger predictor of cardiovascular events than brachial blood pressure.

Together, these studies provide evidence to support the hypothesis that central aortic pressure may more accurately reflect the load on the central vasculature than brachial blood pressure (Table 1) [11–13, 6•, 7]. It is therefore a reasonable proposition that central pressure relates more directly to target organ damage and clinical cardiovascular disease. This has led to the suggestion that central and not brachial blood pressure should be a treatment target for cardiovascular disease risk reduction strategies [7]. The mechanisms by which some antihypertensive drugs, including calcium channel blockers, affect the vasculature and lead to differential lowering of central and brachial blood pressure may be because of their effects on endothelial function, as will now be discussed in more detail.

## Endothelial Dysfunction and the Role of NO

Endothelial dysfunction, a key feature of hypertension, is primarily caused by enhanced oxidative stress, but other important contributors include age, vascular injury, metabolic disorders, deficiencies in essential substrates (e.g., L-arginine), and enzyme cofactors (e.g., tetrahydrobiopterin [BH_4_]) [15]. Endothelial dysfunction is characterized by reduced nitric oxide (NO) bioavailability resulting in increased vascular resistance and reduced sensitivity to normal stimuli of vasodilation, such as shear stress and acetylcholine [16].

The signaling molecule NO is produced by the endothelium and has a key role in regulating vasomotor tone. In addition, NO has atheroprotective effects by reducing smooth muscle cell proliferation and migration, adhesion of leukocytes to the endothelium, and platelet aggregation [17]. NO is derived from the conversion of L-arginine to L-citrulline by the enzymatic activity of endothelial NO synthase (eNOS). The activity of this electron transport enzyme requires calcium/calmodulin, flavin adenine dinucleotide, flavin mononucleotide, and BH_4_ as cofactors.

Normal physiologic levels of NO increase vasodilation and interfere with the atherothrombotic process, thereby helping to maintain a healthy circulatory system. The net concentration of NO in the circulation is dependent on a balance between the enzymatic production of NO through the activity of eNOS and the production of superoxide (O_2_^–^) [17].

**Fig. 1:**
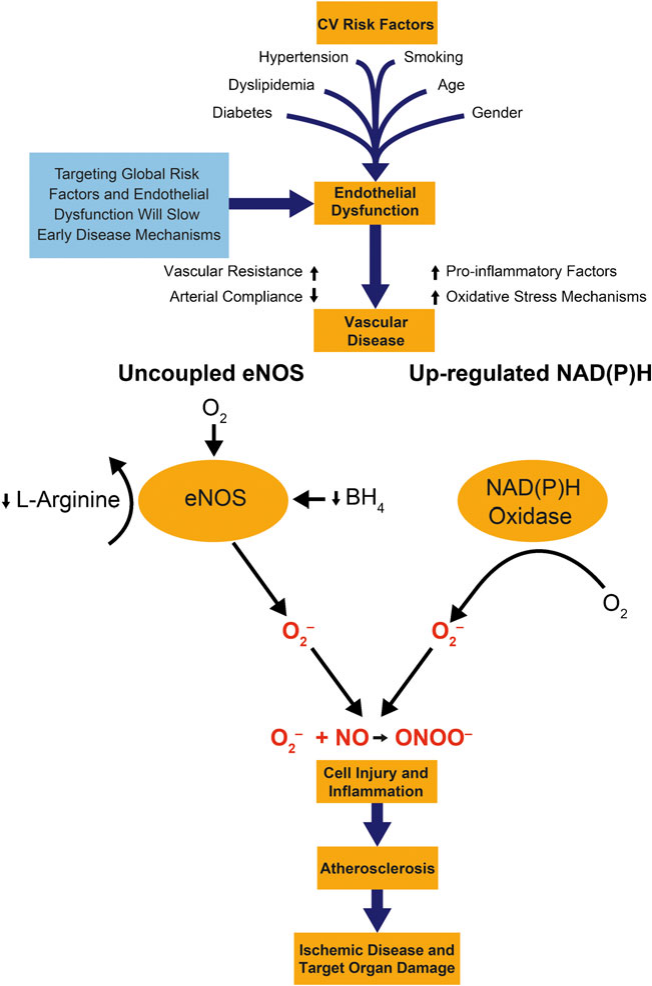
Targeting mechanisms – a global approach to cardiovascular risk management. *eNOS,* endothelial nitric oxide synthase; *BH*_*4*_, tetrahydrobiopterin; *FAD*, flavin adenine dinucleotide; *FMN*, flavin mononucleotide; Ca^2+^, calcium; *O*_*2,*_ oxygen*; O*_*2*_^*–*^, superoxide, NO, nitric oxide; *ONOO* ^*–*^, peroxynitrite

Many factors can influence eNOS activity, but studies have shown that its enzymatic cofactor BH_4_ has a particularly important role [18–20]. When levels of BH_4_ are insufficient eNOS cannot couple the reduction of molecular oxygen with the oxidation of L-arginine. This results in the generation of O_2_^–^ rather than NO [18–20]. This process is known as “eNOS uncoupling” (Fig. 1). Oxidative modification of BH_4_ by various oxidases is a leading reason for abnormal low levels in the cell.

Reactive oxygen species (ROS) are also generated in the vasculature by oxidases such as NAD(P)H oxidase that contribute to oxidative stress [19]. In the presence of excessive levels of O_2_^–^, NO is rapidly converted to peroxynitrite (ONOO^–^) resulting in decreased NO bioavailability and further impairment of endothelium-mediated vasodilation. Furthermore, ONOO^–^ molecules themselves are highly reactive and oxidize lipids, cause cellular injury, and enhanced arterial contraction (Fig. 1) [21•].

Animal models have shown that mice deficient in ROS-generating enzymes have lower blood pressure levels compared with wild-type animals [22]. Further studies found that, compared with normotensive rats, spontaneously hypertensive rats had lower NO bioavailability despite increased levels of eNOS [23]. The effects were even more pronounced after induction of diabetes among the hypertensive animals [21•]. The results were consistent with studies in rats that developed hypertension after aortic banding [24]. The findings of reduced NO bioavailability, despite increased levels of eNOS observed in these studies, may be explained by the increased production of O_2_^–^ by uncoupled eNOS. Glucose intolerance is also believed to impair eNOS activity directly through enhanced oxidative stress.

In a human tissue study comparing endothelial cells from healthy African American and Caucasian donors, those of African American origin were found to have lower levels of NO despite higher levels of eNOS [25]. This paradox was attributed to excessive O_2_^–^ generation by NAD(P)H oxidase, which contributes to increased ONOO^–^ formation and uncoupled eNOS activity [25]. These results are consistent with studies reporting differences in endothelial-dependent vasodilation in African American subjects compared with age- and gender-matched Caucasians [26].

Reduced NO bioavailability may partly explain the higher rates of hypertension observed among African Americans compared with Caucasians in various surveys [27]. This has clinical importance as drug treatments that enhance endothelial NO production may be of particular benefit in these populations.

The finding of a relationship between reduced endothelial-derived NO and increased oxidative stress led to the design of the A-HeFT study (African American Heart Failure Trial) [28]. That trial showed that the addition of isosorbide dinitrate and hydralazine to conventional therapy reduced relative 1-year mortality by 43 % among African American subjects with advanced heart failure [28]. Isosorbide dinitrate is an organic nitrate that directly increases vascular NO levels, whereas hydralazine is a vasodilator with antioxidant activity that may scavenge oxyradical species, including O_2_^–^. This adds further support to the theory that agents that enhance NO bioavailability while reducing nitroxidative stress may have important benefits in this population.

In summary, the relationship between NO and ROS is strongly implicated in the etiology of hypertension, and drugs that have both antihypertensive and antioxidant properties may be more effective at reducing blood pressure and subsequent pathology. Indeed, improving NO bioavailability may be an important treatment goal in the management of hypertension (Fig. 1).

## Effect of Calcium Channel Blockers on Endothelial Function

Dihydropyridine (DHP)-type calcium channel blockers (CCBs) reversibly inhibit calcium entry into cardiac and vascular smooth muscle cells by binding to L-type voltage-sensitive calcium channels [29]. This decreases intracellular calcium concentrations, resulting in smooth muscle cell relaxation. In addition, CCBs have several pleiotropic effects. Of particular importance, certain DHP-type CCBs have been shown to modify endothelial function by enhancing eNOS activity, resulting in increased NO production [29, 30]. Studies have also suggested that some of these drugs increase the antioxidant capacity of the endothelium by scavenging O_2_^–^ [29, 30]. This further protects the endothelium by reducing the availability of free radicals to react with NO. The antioxidant activity is attributed to CCBs’ high lipophilicity, and a chemical structure that facilitates proton-donation and resonance-stabilization mechanisms that inhibit the free-radical reaction [30, 31].

In animal models, the CCB amlodipine has been shown to increase eNOS activity and its mRNA level in hypertensive rats [32]. In coronary microvessels isolated from canine cardiac tissue, amlodipine caused a dose-dependent release of nitrite, the hydration product of NO [33•]. The effects of amlodipine on both nitrite release and the NO-dependent regulation of cardiac oxygen consumption were inhibited with specific antagonists of eNOS such as L-N^G^-monomethylarginine (L-NMMA). Under identical conditions, other DHP and non-DHP-type CCBs, including nifedipine and diltiazem, failed to reproduce these effects.

In clinical studies among patients with essential hypertension, nifedipine has been shown to attenuate circulating plasma levels of lipoperoxides and isoprostanes, increase plasma antioxidant capacity, and restore NO bioavailability [34]. In the Elevation of Nifedipine and Cerivastatin on Recovery of Endothelial Function (ENCORE) I and ENCORE II studies, nifedipine significantly improved NO-mediated coronary endothelial function in patients with coronary artery disease [35, 36].

The safety and efficacy of CCB therapy and its role in reducing cardiovascular events and procedures has been demonstrated in several large clinical studies among patients with and without established cardiovascular disease [37–46, 47•, 48•]. Key findings from these studies are summarized in Table 2 for both DHP-type CCBs and other members of this drug class.

**Table 2:** Outcome trials using calcium channel blockers

Study	Design/drug	Patient population	Key result
ACCOMPLISH (2008) [48•]	Double-blind, randomized trial. Benazepril + amlodipine or benazepril + hydrochlorothiazide. Mean follow-up 36 months.	11,506 patients with hypertension at high risk of CV events.	Compared with benazepril + hydrochlorothiazide, fewer individuals on benazepril + amlodipine had a primary endpoint (death from CV causes, nonfatal MI, nonfatal stroke, hospitalization for angina, resuscitation after sudden cardiac arrest, and coronary revascularization) HR, 0.80; 95 % CI, 0.72 to 0.90; *P* < 0.001. For the secondary endpoint of death from CV causes, nonfatal MI, and nonfatal stroke, HR 0.79; 95 % CI, 0.67 to 0.92; *P* = 0.002.
ASCOT-BPLA (2005) [47•]	Open-label, randomized trial. Amlodipine ± perindopril-based regimen or atenolol ± bendroflumethiazide-based regimen. Mean 5.5 year follow-up.	19,257 patients with hypertension and ≥3 additional CV risk factors.	Compared with the atenolol-based regimen, fewer individuals on the amlodipine-based regimen had a primary endpoint (nonfatal MI and fatal CHD) HR, 0.90; 95 % CI, 0.79 to 1.02; *P* = 0.1052; fatal and nonfatal stroke, HR, 0.77; 95 % CI, 0.66 to 0.89; *P* = 0.0003; total CV events and procedures, HR, 0.84; 95 % CI, 0.78 to 0.90; *P* < 0.0001; all-cause mortality, HR, 0.89; 95 % CI, 0.81 to 0.99; *P* = 0.025.
CAMELOT (2004) [46]	Double-blind, placebo-controlled, randomized trial. Amlodipine, enalapril, or placebo. 24 month follow-up.	1,991 patients with CAD and DBP <100 mm Hg.	Compared with placebo, there was a 31 % reduction in CV events in the amlodipine group (*P* = 0.003) and a 15 % reduction in the enalapril group (*P* = 0.16). In the amlodipine group, IVUS showed evidence of slowing atherosclerosis progression.
VALUE (2004)[45]	Double-blind, parallel-group, randomized trial. Valsartan or amlodipine. Mean follow-up 4.2 years.	15,245 patients with hypertension at high risk of cardiac events.	No difference in the primary outcome (cardiac mortality and morbidity) between treatment groups, HR 1.04; 95 % CI, 0.94 to 1.15; *P* = 0.49. BP reduced by both treatments, but amlodipine had greater effect especially in the early period. Amlodipine was superior to valsartan at preventing MI and angina.
INVEST (2003)[44]	Open-label, blinded endpoint, randomized trial. Verapamil or atenolol. Mean follow-up 2.7 years.	22,576 patients with hypertension and CAD.	No difference in the primary outcome (first occurrence of all-cause mortality, nonfatal MI or nonfatal stroke) between treatment groups, RR 0.98; 95 % CI 0.90-1.06.
CONVINCE (2003)[43]	Double-blind, randomized trial. Verapamil versus atenolol or hydrochlorothiazide. Mean follow-up 3 years.	16,602 patients with hypertension and ≥1 additional CV risk factor.	No difference in the primary outcome (first occurrence of stroke, MI, or CV-related death) between treatment groups, HR 1.02; 95 % CI, 0.88 to 1.18; *P* = 0.77.
ALLHAT (2002)[42]	Double-blind, randomized trial. 3 treatment groups: chlorthalidone; amlodipine; lisinopril. Mean follow-up 4.9 years.	33,357 patients with hypertension and ≥1 additional CHD risk factor.	No difference in the primary outcome (fatal CHD or nonfatal MI) between treatment groups. Compared with chlorthalidone: RR for amlodipine 0.98; 95 % CI, 0.90 to 1.07; lisinopril 0.99; 95 % CI, 0.91 to 1.08.
NORDIL (2000)[40]	Open-label, blinded endpoint, randomized trial. Diltiazem or diuretics ± beta-blockers. Mean follow-up 4.5 years.	10,881 patients with DBP ≥100 mm Hg.	No difference in the primary outcome (fatal and non-fatal stroke, MI, CV death) between the 2 groups, RR 1.00; 95 % CI, 0.87 to 1.15; *P* = 0.97.
INSIGHT (2000)[39]	Double-blind, randomized trial. Nifedipine or co-amilozide. Follow-up 3 years after recruitment of the last patient.	6,321 patients with hypertension and ≥1 additional CV risk factor.	No difference in the primary outcome (CV death, MI, heart failure, or stroke) between the 2 groups, RR, 1.10; 95 % CI, 0.91 to 1.34; *P* = 0.35.
PREVENT (2000) [41]	Double-blind, placebo-controlled, randomized trial. Amlodipine or placebo. 36-month follow-up.	825 patients with CAD.	No difference in coronary stenosis between the amlodipine and placebo group. Amlodipine slowed the progression of carotid artery atherosclerosis (IMT: amlodipine −0.0126 versus placebo +0.033; *P* = 0.007) and was associated with fewer hospitalizations for unstable angina and coronary revascularization.
SYST-EUR (1997) [38]	Double-blind, placebo-controlled, randomized trial. Nitrendipine or placebo. Median follow-up 2 years.	4695 patients with isolated systolic hypertension (SBP ≥160 mmHg and DBP <95 mmHg).	Compared with placebo, nitrendipine reduced the total rate of stroke by 42 % (*P* = 0.003); nonfatal stroke by 44 % (*P* = 0.007) and all fatal and nonfatal cardiac events by 26 % (*P* = 0.03).
MIDAS (1996) [37]	Double-blind, randomized trial. Isradipine or hydrochlorothiazide. 3 year follow-up.	883 patients with hypertension	No difference in the rate of progression of mean maximum IMT (*P* = 0.68) between treatment groups. Higher (but non-significant, *P* = 0.07) incidence of major vascular events (MI, stroke, heart failure, angina, sudden death) in the isradipine versus the hydrochlorothiazide group (25 vs 14 events).

*ACCOMPLISH* Avoiding Cardiovascular Events through Combination Therapy in Patients Living with Systolic Hypertension study; *ASCOT-BPLA* Anglo-Scandinavian Cardiac Outcomes Trial – Blood Pressure Lowering Arm; *CAMELOT* Comparison of Amlodipine versus Enalapril to Limit Occurrences of Thrombosis study; *VALUE*, Valsartan Antihypertensive Long-term Use Evaluation; *INVEST*, The International Verapamil-Trandolapril Study; *CONVINCE*, Controlled Onset Verapamil Investigation of Cardiovascular End Points; *ALLHAT*, The Antihypertensive and Lipid-Lowering Treatment to Prevent Heart Attack Trial; *NORDIL*, the Nordic Diltiazem study; *INSIGHT*, Intervention as a Goal in Hypertension Treatment; *PREVENT* Prospective Randomized Evaluation of the Vascular Effects of Norvasc Trial; *SYS-EUR*, Systolic Hypertension in Europe; *MIDAS*, Multicenter Isradipine Diuretic Atherosclerosis Study.

*CAD* coronary artery disease; *CHD* coronary heart disease; *CI* confidence interval; *CV* cardiovascular; *DBP* diastolic blood pressure; *HR* hazard ratio; *IMT* intimal-media thickness; *IVUS* intravascular ultrasound; *MI* myocardial infarction; *SBP*, systolic blood pressure

## Role of Combination Therapy in Cardiovascular Risk Management

Hypertension commonly clusters with other cardiovascular risk factors, including dyslipidemia and diabetes, greatly increasing an individual’s risk of an event [49, 50]. From a mechanistic viewpoint, these risk factors act synergistically to exacerbate endothelial dysfunction, oxidative stress, and inflammation, thereby accelerating the atherosclerotic process [51].

Although controversy remains over the optimal choice of antihypertensive drug therapy, hypertension management guidelines agree that most patients may require at least two antihypertensive drugs to reach the currently recommended blood pressure targets [52–54]. In addition, many patients with hypertension also benefit from concurrent statin therapy [52–54]. Thus most patients with hypertension require multiple drug treatment regimens to manage their cardiovascular risk.

A greater understanding of the mechanisms of actions of antihypertensive and lipid-lowering drugs may lead to more effective drug combinations with respect to clinical outcomes. This review focuses on the combination of CCBs with statins and CCBs in combination with drugs that affect the renin-angiotensin system (RAS).

## Rationale for Combining Calcium Channel Blockers and Statins

Both CCBs and statins have been shown to reduce cardiovascular events in large clinical outcome trials [45, 46, 55, 56, 47•, 48•]. ASCOT highlighted a potential synergy between co-administered amlodipine and atorvastatin in terms of the cardio-protective effect of this drug combination [57]. In ASCOT, those randomized to amlodipine plus atorvastatin had a 53 % reduction in coronary heart disease events compared with a 16 % reduction among those randomized to atenolol plus atorvastatin. This could not be explained by differences in blood pressure and lipid parameters between the two treatment regimens. Furthermore, the significant benefits of the amlodipine plus atorvastatin combination were observed within the first 3 months of treatment (*P* = 0.02) [57]. This is suggestive of a functional rather than a structural change to the vasculature.

The findings from ASCOT [57] may be explained, in part, by observations from the AVALON Arterial Wall Compliance (AWC) trial [58]. In the AWC trial, 668 patients (61 % male, mean age 55 years) with concomitant hypertension and dyslipidemia were randomized to one of four treatment groups (placebo, amlodipine 5 mg, atorvastatin 10 mg, co-administered amlodipine 5 mg and atorvastatin 10 mg) [58]. Arterial compliance was assessed every 4 weeks. After 8 weeks of treatment, there was a 19.3 % improvement in small artery compliance in the co-administered amlodipine and atorvastatin group compared with 11.7 % in the amlodipine alone group (*P* = 0.03), 3.1 % in the atorvastatin alone group (*P* < 0.001), and −1.3 % in the placebo group (*P* < 0.0001). After 28 weeks of treatment, the greatest improvement in small artery compliance remained among those taking co-administered amlodipine and atorvastatin (*P* < 0.05) [58]. The observation that combination therapy had a more than additive effect on small artery compliance is consistent with an improvement in the mechanisms that control vascular function [59•]. Indeed, the authors of the AWC trial proposed that their study provided evidence that the synergistic effects of co-administered amlodipine and atorvastatin may be mediated by endothelial function [58].

This hypothesis is supported by the results of the ENCORE I study that compared NO-mediated endothelial function among 343 patients with coronary artery disease [35]. Patients were randomized to 6 months treatment with placebo, cerivastatin 0.4 mg/day, nifedipine 30 to 60 mg/day, or their combination. Endothelial function was assessed by infusing acetylcholine into a coronary segment and measuring luminal diameter by quantitative angiography. In the most constricted segment, nifedipine but not cerivastatin, significantly reduced acetylcholine-induced vasoconstriction by 18.8 % (*P* < 0.05, compared with placebo). The combination of nifedipine and cerivastatin also reduced acetylcholine-induced vasoconstriction by 11 %, but this only reached statistical significance when all coronary segments were analyzed together. After cerivastatin was withdrawn from the market in 2001, the ENCORE study design was modified to compare nifedipine with placebo [36]. In ENCORE II, all patients were eligible for statin therapy at the discretion of their physician. In addition to measuring changes in luminal diameter, intravascular ultrasound (IVUS) was used to assess change in plaque volume. Overall, 454 patients were randomized and followed for 18–24 months. Compared with placebo, nifedipine plus background statin therapy significantly improved coronary endothelial function in the most constricted segment (difference in luminal diameter 6.3 %, 95 % CI 1.6 to 10.9; *P* = 0.0088). However, compared with placebo, nifedipine plus background statin therapy did not have a significant effect on coronary plaque volume as measured by IVUS. Together the ENCORE studies add to the evidence that CCB plus statin therapy improves NO-mediated endothelial function [35, 36].

From a mechanistic perspective, synergy between CCBs and statins is plausible, as these drugs have complementary chemical structures. Atorvastatin has negative polarity associated with its heptanoic side chain, whereas amlodipine is distinct among the DHPs in having a positive charge on its aminoethoxy side chain [31, 60, 61]. Both amlodipine and atorvastatin are lipophilic and share high affinity for the cell membrane. Indeed, studies have demonstrated that the concentration of these drugs is much higher in the cell membrane than in the surrounding aqueous environment [31, 60, 61]. These properties may facilitate interactions with novel receptor sites in vascular cell membranes, and contribute to explaining the superior clinical outcomes observed when these drugs are co-administered. This hypothesis was evaluated in a study using human umbilical vein endothelial cells (HUVEC) [59•]. The combination of amlodipine and atorvastatin directly stimulated NO release, which was about twofold greater than the sum of their separate effects (*P* < 0.05). This was attributed to enhanced eNOS function and expression along with decreased levels of cytotoxic ONOO^–^. Following low density lipoprotein (LDL) enrichment there was a 60 % reduction in NO production in the HUVEC and an almost twofold increase in ONOO^–^. Treatment with the combination of amlodipine and atorvastatin partially reversed the adverse effects of LDL, including a 90 % increase in NO and a 50 % reduction in ONOO^–^. Small angle X-ray diffraction analysis indicated that both amlodipine and atorvastatin are lipophilic and share a common membrane location [59•]. This study provides evidence to suggest the observed synergy between these drugs may be explained by electron transport mechanisms that facilitate antioxidant activity in the cell membrane related to their complementary locations.

## Rationale for Combining CCBs and Drugs that Affect the RAS

The RAS has an important role in blood pressure control by regulating blood volume and peripheral vascular resistance. In brief, in response to the release of renin from the kidneys, the circulating substrate angiotensinogen is converted to angiotensin I. The angiotensin-converting enzyme (ACE) in the vascular endothelium cleaves off two amino acids to form the octapeptide, angiotensin II (Ang II). Ang II binds to angiotensin II type-1 (AT_1_) receptors in vascular smooth muscle cells, promoting vasoconstriction and increasing peripheral vascular resistance. Ang II also stimulates the release of various hormones including aldosterone and vasopressin, which act on the kidneys to increase sodium and fluid retention. Ang II also facilitates norepinephrine release from sympathetic nerve endings and inhibits its reuptake, thereby enhancing sympathetic adrenergic function. The interaction between Ang II and AT_1_ receptors also activates signal transduction mechanisms that promote oxidative stress, inflammation, cell proliferation, and fibrosis [62]. Studies have shown that Ang II activates NAD(P)H oxidase in endothelial and vascular smooth muscle cells. This results in the production of O_2_^–^ and other ROS, and contributes directly to reduced NO bioavailability and endothelial dysfunction [63].

Blocking the RAS with ACE inhibitors and angiotensin receptor blockers (ARBs) has been demonstrated to improve endothelial function and increase NO bioavailability in mechanistic [64–67] and clinical [68–70] studies.

In an animal model using infarcted adult male Sprague–Dawley rats, treatment with the ARB candesartan, enhanced vasorelaxation by increasing NO bioavailability via an AT_2_ receptor mediated upregulation of eNOS [64]. A complementary in vitro study, using the ARBs losartan and valsartan, stimulated NO release from both platelets and human umbilical vein endothelial cells in a dose-dependent manner. However, there was more than 70 % greater potency in NO release in platelets than endothelial cells. Furthermore, the degree of inhibition of platelet adhesion and aggregation by losartan and valsartan was closely correlated with the level of NO production [65]. In an animal model using isolated coronary arterioles from pigs, Ang II was shown to evoke AT_1_ receptor-mediated vasoconstriction and AT_2_ receptor-mediated vasodilation. At the vascular level, Ang II was shown to impair endothelium-dependent NO-mediated dilation attributable to elevated O_2_^–^ production via AT_1_ receptor activation of NAD(P)H oxidase. The authors of this study commented that their results may partly explain why impaired coronary flow is associated with upregulation of the RAS [66]. The long-term effect of the ARB valsartan on endothelial function and vascular structural changes in the aorta was explored in hypercholesterolemic rabbits. Treatments with 3 and 10 mg/kg per day of valsartan reduced intimal lesion to 2.4 ± 0.7 % and 2.7 ± 0.9 %, respectively (*P* < 0.05) and increased lumen area. The authors suggested that AT_1_ receptor antagonists, besides their antihypertensive effects, could also have a role in reducing the development of atherosclerosis [67].

The results of these mechanistic studies were consistent with the results from clinical studies [68–70]. In an active-controlled, randomized trial, 35 patients with coronary artery disease received 4 weeks of treatment with either an ACE inhibitor (ramipril 10 mg/day) or ARB (losartan 100 mg/day). NO-mediated vasodilation of the radial artery was determined before and after intra-arterial L-NMMA infusion. NO-mediated vasodilation was increased by more than 75 % after treatment with ramipril and losartan (each group *P* < 0.01) [68]. These results were consistent with a double-blind, placebo-controlled trial among 60 patients with essential hypertension who received 6 weeks of treatment with either an ARB (valsartan 80 mg/day), a diuretic (hydrochlorothiazide 25 mg/day), or placebo. NO production was assessed using forearm blood flow techniques. There were similar reductions in brachial blood pressure in both active treatment arms (*P* < 0.001). However, patients in the ARB group had significantly improved vasoconstrictive response to L-NMMA, whereas no effect was seen in the diuretic and placebo groups [69]. Together, these clinical studies suggest that agents that block the RAS may improve endothelial function by increasing NO bioavailability. A further study, among 53 hypertensive patients with and without type 2 diabetes, showed that treatment with valsartan 80 mg/day for 8 weeks significantly decreased monocyte and endothelial cell activation markers among those with diabetes, but not among those without diabetes [70]. This study highlights the importance of using complex disease models to explore mechanisms of actions, because focusing on single-risk-factor models may miss important biological pathways.

There is a growing body of evidence to suggest that DHP-type CCBs and RAS inhibitors have additional beneficial effects when used in combination. For example, in an animal model using Dahl salt-sensitive rats, the combination of CCB plus ACE inhibitor was more effective than either monotherapy in normalizing systolic blood pressure and proteinuria [71]. This study was complimented by a rat myocardial infarction model that showed that treatment with the CCB amlodipine plus ACE inhibitor benazepril increased NO production and decreased inflammatory markers. In contrast, treatment with the diuretic hydrochlorothiazide had no effect on these indicators of endothelial dysfunction [72].

In clinical studies, the combination of CCBs with an ACE inhibitor versus a CCB alone has been shown to increase flow-mediated dilation, a surrogate biomarker of endothelial function [73]. As previously described, the CAFE study [6•] demonstrated that the benefits of the CCB plus ACE inhibitor combination may extend beyond brachial blood pressure lowering and have a positive impact on central blood pressure hemodynamics [6•]. Furthermore, outcome trials have reported that these beneficial effects may translate into reduced cardiovascular endpoints. The ASCOT-BPLA study showed that treatment with an amlodipine plus perindopril-based regimen prevented more major cardiovascular events than an atenolol plus bendroflumethiazide-based regimen [47•]. The ACCOMPLISH study showed that treatment with benazepril plus amlodipine was more effective at preventing cardiovascular events than treatment with benazepril plus hydrochlorothiazide [48•].

Together, these mechanistic and clinical studies demonstrate the pleiotropic effects of the combination of CCB plus RAS blockade on enhanced NO bioavailability and reduced oxidative stress. Understanding the complementary mechanisms of actions of these drugs forms the rationale for combining different antihypertensive agents to enhance their therapeutic effects.

## Conclusions

This review has explored the pleiotropic effects of CCBs alone and in combination with statins and RAS inhibitors. The mechanisms by which these drugs exert their beneficial effects on endothelial and vascular function have been described, with a particular focus on the effects on NO-mediated vasodilation and oxidative stress. By further elucidating the biological pathways by which these drugs exert their effects, we considered the rationale for choosing optimal drug combinations that will benefit patients at risk. The differential effect of CCBs on brachial and central blood pressure has been explored, along with the future role of central blood pressure as a prognostic marker for cardiovascular disease events. Indeed, clinical hypertension trials have demonstrated the importance of incorporating measurements of central aortic pressure in their study designs. This will provide much needed outcome data to support evidence-based practice. Another important aspect of future hypertension research is the shift towards studying complex disease models. From a mechanistic viewpoint, multiple cardiovascular risk factors act synergistically to exacerbate endothelial dysfunction, oxidative stress and inflammation, thereby accelerating the atherosclerotic process. It is therefore logical that they should be considered together in global risk assessment. By further elucidating the underlying biological pathways and mechanisms that lead to target organ damage in multiple cardiovascular risk factor models, we may be able to define new therapeutic targets and develop novel treatments. Such approaches will continue to require the scientific community to work together to carry out large-scale studies and to link the findings from experimental models with human populations.
